# Graphene Oxide/Polyvinyl Alcohol/Fe_3_O_4_ Nanocomposite: An Efficient Adsorbent for Co(II) Ion Removal

**DOI:** 10.1155/2021/6670913

**Published:** 2021-03-09

**Authors:** Thu Dieu Le, Luyen Thi Tran, Hue Thi Minh Dang, Thi Thu Huyen Tran, Hoang Vinh Tran

**Affiliations:** School of Chemical Engineering, Hanoi University of Science and Technology, 1 Dai Co Viet Road, Hanoi, Vietnam

## Abstract

In this work, an effective nanocomposite-based adsorbent directed to adsorb cobalt (Co^2+^) ion was successfully synthesized from graphene oxide (GO), polyvinyl alcohol (PVA), and magnetite (Fe_3_O_4_) nanoparticles via a coprecipitation technique. The synthesized GO/PVA/Fe_3_O_4_ nanocomposite was applied for Co^2+^ ion removal with the optimized working conditions including 100 min of contact time, 0.01 g of adsorbent dosage, pH of 5.2, and 50°C of temperature. The investigation of adsorption kinetics showed that the adsorption of Co^2+^ ion onto the GO/PVA/Fe_3_O_4_ nanocomposite followed the pseudo-second-order kinetic model with the rate constant k_2_ being 0.0026 (g mg^−1^·min^−1^). The Langmuir model is suitable to describe the adsorption of Co^2+^ ion onto the GO/PVA/Fe_3_O_4_ nanocomposite with the maximum sorption capacity (*q*_max_) reaching 373.37 mg·g^−1^. The obtained results also indicated that the GO/PVA/Fe_3_O_4_ nanocomposite can adsorb/regenerate for at least 5 cycles with a little reduction in removal efficiency. Therefore, we believe that the GO/PVA/Fe_3_O_4_ nanocomposite could be used as a potential adsorbent for heavy metal treatment in terms of high adsorption capacity, fast adsorption rate, and recyclability.

## 1. Introduction

Nowadays, electronic devices are becoming more and more common in our life, in which rechargeable batteries are an indispensable item in every family and for every individual. However, the reality indicates that the manufacturing of rechargeable batteries, electrodes, gas turbine engines, hard permanent magnets etc. use a huge amount of cobalt (Co) and discharge a lot of cobalt ions (Co^2+^) into the environment. Inhalation of Co dust may cause adverse respiratory effects, also causing neurological symptoms and cancer in human beings with unknown mechanism [1–3]. Therefore, there are many kinds of technologies to reduce the concentration of Co^2+^ in water pollutants, such as nanofiltration, adsorption, and ion exchange, in which the adsorption process is the best choice because it is cheap and suitable to adapt with a vast range of working conditions to remove Co^2+^ ion from aqueous solutions [4–10]. In order to improve the adsorption efficiency, some advanced nanomaterials have been applied for enhancing the specific surface area of the adsorbent which favors adsorption using carbon nanotubes (CNTs) [11–13], activated carbon [14, 15], graphite [16], graphene oxide (GO) [17], or reduced graphene oxide [18]. These nanomaterials have many functional groups on the surface such as −COOH, −OH, and C=O which can be used as an electron-trapping site to attract metal ions or organic materials [11–14, 17]. Recently, GO is widely applied as an adsorbent directed to adsorb heavy metal ions from water because GO has a large surface area (which can be up to 2630 m^2^·g^−1^) and high water solubility [19–22]. In addition, GO has abundant oxygen-based groups on its surface such as hydroxyl, carboxylic, carbonyl, and epoxide groups, making GO a material of great interest in adsorption-based technologies of water treatment. GO adsorbents with excellent maximum adsorption capacity (*q*_max_) were reported, such as *q*_max_ = 198 mg·g^−1^ for adsorption of Cr(VI) ion [23] and the *q*_max_ = 46.6 mg·g^−1^ for adsorption of Cu(II) ion [24]. However, GO is a nano/micromaterial and it has very low density; therefore, it is difficult to remove GO out of the water after adsorption processes. The hybrids of graphene with magnetic nanomaterials such as Fe_3_O_4_ nanoparticles have been used to solve the above problem. Fe_3_O_4_ is usually used for water purification due to its safety; in addition, the Fe_3_O_4_ is also used to generate magnetic properties for the adsorbent, which makes it easy to be collected after treatment by using an external magnet bar. Yao et al. [25] reported Fe_3_O_4_@graphene in dye removal with *q*_max_ of 45.27 mg·g^−1^ to methylene blue (MB) and 33.66 mg·g^−1^ to Congo red [25]. Uheida et al. [26] has used Fe_3_O_4_ and ɤ-Fe_2_O_3_ nanoparticles for the removal of Co^2+^ ion. To improve the bonding of Fe_3_O_4_ nanoparticles with the GO sheets, a natural polymer or synthetic polymer can be used. In fact, polyvinyl alcohol (PVA) is widely used in the adsorption process because of its nontoxicity, low cost, and chemical stability and having many hydroxyl (-OH) groups [27, 28]. Wang et al. [28] have fabricated the GO-PVA composites and showed that GO-PVA can adsorb MB with a *q*_max_ of 571.4 mg·g^−1^. We have reported the use of GO/chitosan/Fe_3_O_4_ nanocomposite as a recoverable and recyclable adsorbent for Cr(IV) ion adsorption with easy removal of the GO/chitosan/Fe_3_O_4_ composite adsorbent out of the solution by using a magnetic bar and especially high adsorption capacity (*q*_max_ = 200 mg·g^−1^) for Cr(IV) ion [29]. In this study, we extend the above approach with chitosan being replaced by PVA for synthesis of the GO/PVA/Fe_3_O_4_ composite, which was directed to adsorb of Co^2+^ ion.

## 2. Experimental

### 2.1. Materials and Reagents

Concentrated sulfuric acid (H_2_SO_4_ 98 wt.%), ammonium iron (II) sulfate hexahydrate ((NH_4_)_2_Fe(SO_4_)_2_.6H_2_O, 99 wt.%), ethanol (C_2_H_5_OH, 96 v/v.%), and hydrochloric acid (HCl) were purchased from Duc Giang Chemical Co., Ltd. (Vietnam). Potassium sulfate (K_2_SO_4_, 99 wt.%), iron(III) chloride hexahydrate (FeCl_3_.6H_2_O, 99 wt.%), sodium hydroxide (NaOH, 99 wt.%), and acetic acid (CH_3_COOH, 99 wt.%) were purchased from Xilong (China). Polyvinyl alcohol (PVA) (*M*_w_ ≈ 47 000, 87–90% hydrolyzed), ammonium thiocyanate (NH_4_SCN, ≥wt.%), and acetone were purchased from Sigma-Aldrich. Aqueous ammonia is an analytical reagent and used without further purification. GO was purchased from Graphitene Ltd., and CoCl_2_.6H_2_O (≥99 wt.%) was purchased from Merck.

### 2.2. Preparation of Fe_3_O_4_ Nanoparticles

Fe_3_O_4_ nanoparticles were prepared by the coprecipitation method. First, 0.951 g of FeCl_3_.6H_2_O and 0.69 g of (NH_4_)_2_Fe(SO_4_)_2_.6H_2_O were dissolved in 50 ml of distilled water. Then, 10 wt.% ammonia solution is added drop by drop to adjust pH of the solution to 8-9. The black precipitate of Fe_3_O_4_ will be obtained.

### 2.3. Preparation of GO/PVA/Fe_3_O_4_ Nanocomposite

0.1 g of GO was ultrasonicated in 30 ml of distilled water at ambient conditions to have a slurry solution. 0.2 g of PVA was dissolved in 30 ml of distilled water and stirred at 500 rpm at 90°C. When all PVA is dissolved completely and GO is dispersed well, they are mixed with a mixture of Fe_3_O_4_ colloid, stirred for 10 minutes and filtered, washed many times with distilled water, and finally dried in an oven at 40°C for 1 day.

### 2.4. Batch Adsorption Experiments for Co^2+^ Ion Removal

0.0406 g of CoCl_2_.6H_2_O was dissolved in 100 mL distilled water to obtain the stock solution of Co^2+^ ion (100 mg·L^−1^). The stock solution was diluted to the desired solution. 0.01 g of GO/PVA/Fe_3_O_4_ nanocomposite as an adsorbent was added into 20 mL of the solution containing Co^2+^ ion; then, the mixtures were agitated at 30°C and pH 7 for 100 min. The residue Co^2+^ ion concentration in the solution was analysed by the spectrophotometric method (described in Section 3.5). The adsorption capacity, *q* (mg·g^−1^), and the percentage removal (H, %) were calculated by the following equations:

(1) $$\begin{array}{c} q=\frac{\left(C_{0}-\;C\right)\cdot V}{m}\;\left(mg\cdot g^{-1}\right),\\ H=\;\frac{\left(C_{0}-C_{e}\right)}{C_{0}}\cdot 100\%\;\left(\%\right), \end{array}$$

where *C*_0_ and *C*_*e*_ are the initial and equilibrium concentrations of Co^2+^ solution (mg·L^−1^), respectively; *C* is the Co^2+^ ion concentration at time *t*; *V* is the volume of the sample solution used for the experiment (mL); and *m* is the weight of the adsorbent (g). The adsorption of Co^2+^ ion onto GO/PVA/Fe_3_O_4_ was studied as a function of contact time, mass of adsorbent influent, pH of the solution, and temperature.

The used GO/PVA@Fe_3_O_4_ adsorbent was recovered by immersing it into 0.1 N NaOH solution for 1 day, and then, it was rinsed with distilled water and dried at 40°C in an oven for 12 h to obtain the regenerated GO/PVA@Fe_3_O_4_ adsorbent, which was reused to adsorb Co^2+^ ion.

### 2.5. Determination of Co^2+^ Ion Concentration

The Co^2+^ ion concentration after adsorption process is determined by the spectrophotometric method with the aid of many complexing reagents, which had been developed recently to monitor Co^2+^ concentration, with fast response, high sensitivity, and easy preparation compared to other methods. The common complexing reagents used include ninhydrin (optimum pH is 8.2; the complex is stable in 30 min) [30], 2-benzoylpyridine-4-phenyl-3-thiosemicarbazone [31], 2-pyridine carboxaldehyde isonicotinyl hydrazone (pH 9; the apparent molar adsorptivity is 7.1.10^4^ L·mol^−1^·cm^−1^) [32], dehydroacetic acid oxime (pH 5.8) [33], and 5-[3-(1,2,4-triazolyl-azo]-2,4-dihydroxybenzaldehyde [34]. In our work, we used NH_4_SCN as a ligand in acetone solution and we have discovered that it can be stable up to more than 7 days. A typical procedure is followed: 1 ml of 18% HCl solution is added into 10 ml of residue solution at RT. Then, 0.5 ml of NH_4_SCN saturated solution is added into the above mixture and the solution is mixed well. After that, 20 ml of concentrated acetone is added into this mixture and the solution will change from pink color to blue color in the following reaction:

(2) $$\begin{array}{c} {\text{CoCl}}_{2}{+4\text{NH}}_{4}\text{SCN}⟶{\left(\text{NH}4\right)}_{2}\left[\text{Co}\left(\text{SCN}\right)4\right]{+2\text{NH}}_{4}\text{Cl}\\ \left(\text{pink color}\right)\;\left(\text{blue color}\right) \end{array}$$

The absorbance of the mixture is then measured with an Agilent 8453 UV-Vis spectrophotometer, and the calibration curve is obtained (Figure S1). Each sample was measured in duplicate.

### 2.6. Characterizations

XRD patterns of GO and GO/PVA/Fe_3_O_4_ were obtained on D8 Advance, Brucker ASX, operated at a CuK_*α*_ wavelength of 1.542 $\dot{A}$ in the range of 2*θ* = 5 to 70° at the room temperature. UV-Vis spectra were measured with an Agilent 8453 UV-Vis spectrophotometer system. The fracture surfaces of GO and GO/PVA/Fe_3_O_4_ were observed using a Hitachi S4500 Scanning Electron Microscope (SEM). The infrared (IR) spectra were recorded on a Nicolet FT-IR Spectrometer model 205 with KBr pellets in the region from 500 cm^−1^ to 4000 cm^−1^. Specific surface area and pore size distribution of the prepared GO/PVA/Fe_3_O_4_ sample were evaluated using low-temperature nitrogen adsorption isotherm by Brunauer-Emmett-Teller (BET) and Barrett-Joyner-Halenda (BJH) methods on the Tristar II plus System (Micromeritics, USA).

## 3. Results and Discussion

### 3.1. Characterization of the GO/PVA/Fe_3_O_4_ Nanocomposite

**Figure 1:**
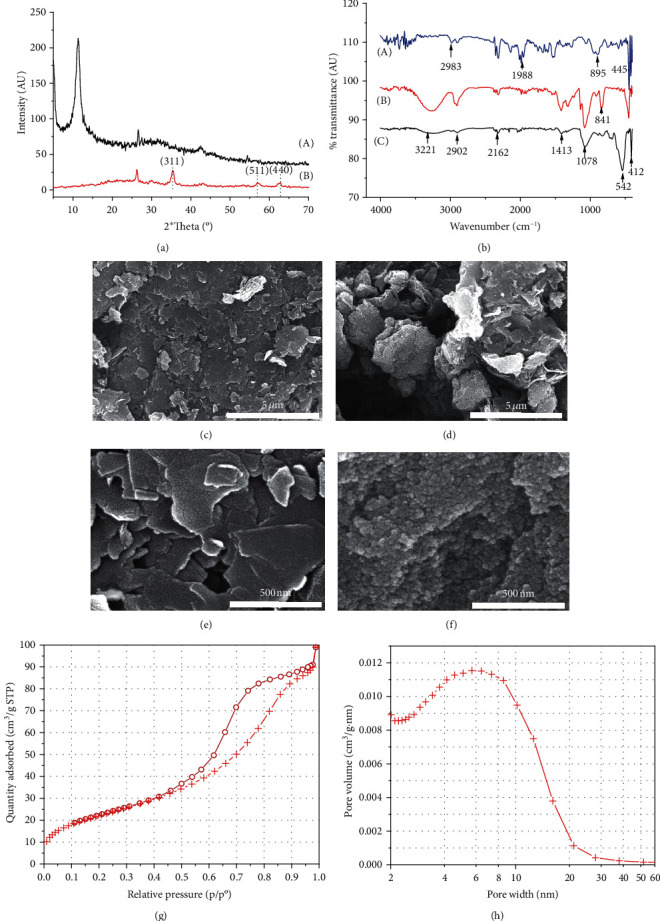
(a) XRD patterns of GO (A) and GO/PVA/Fe_3_O_4_ (B) sample; (b) FT-IR of GO (A), PVA (B), and GO/PVA/Fe_3_O_4_ (C); (c–f) SEM images with different magnifications of (c, e) GO and (d, f) GO/PVA/Fe_3_O_4_, respectively; nitrogen (N_2_) adsorption-desorption isotherm (g) and BJH pore size distribution (h) of the as-synthesized GO/PVA/Fe_3_O_4_ sample.

The XRD patterns of GO and GO/PVA/Fe_3_O_4_ (Figure 1(a)) show a diffraction peak at 2*θ* = 10°, which is assigned to the crystalline of GO (curve A) with (001) reflection indicating that the oxygen functionality existence increases the distance between graphene layers. Using Bragg's Law and Scherrer equation for this sharp peak, it is revealed that the interlayer space is about 0.885 nm and the number of layers in GO is 5. In case of GO/PVA/Fe_3_O_4_ (curve b), this peak disappears due to a very low content of GO in the sample; however, the characteristic peaks of Fe_3_O_4_ clearly appeared at 2*θ* = 30°, 35°, 57°, and 63° corresponding to the reflection of (220), (311), (511), and (440), respectively.

The FT-IR spectrum of GO/PVA/Fe_3_O_4_ (Figure 1(b), curve C) shows the band at 3221 cm^−1^, which denotes to the –OH stretching of physisorbed water. The band at 2902 cm^−1^ attributed to the C-H stretching vibration, and the characteristic peaks ascribed to C-OH groups (1413 cm^−1^ and 1078 cm^−1^) in PVA were found in both the FT-IR spectrum of PVA and GO/PVA/Fe_3_O_4_ (Figure 1(b), curve B and curve C, respectively) confirming the presence of PVA in the GO/PVA/Fe_3_O_4_ samples. A peak at 542 cm^−1^ is attributed to the Fe-O group of Fe_3_O_4_ in GO/PVA/Fe_3_O_4_ samples (curve C), indicating that Fe_3_O_4_ is linked successfully to the GO and PVA. SEM images of GO (Figures 1(c) and 1(e)) show that the GO materials are arranged in sheets. SEM images of the GO/PVA/Fe_3_O_4_ adsorbent (Figures 1(d) and 1(f)) show an appearance of spherical particles with a size of about 15–20 nm, deposited on the GO sheets, nearly covering all the surface of GO sheets, making it difficult to see the GO sheets. It can be seen the Fe_3_O_4_ nanoparticles were well distributed on the GO sheets (Figure 1(f)); these Fe_3_O_4_ nanoparticles contribute to making the magnetic property recover the GO/PVA/Fe_3_O_4_ adsorbent from the solution after the adsorption process by using an external magnet. The hysteresis loop of the nitrogen adsorption-desorption isotherm of the GO/PVA/Fe_3_O_4_ nanocomposite (Figure 1(g)) exhibits type IVa hysteresis loops by IUPAC, which is specific to mesoporous materials with a pore width range from 4 to 50 nm [35]. The BJH pore size distribution of GO/PVA/Fe_3_O_4_ sample (Figure 1(h)) shows the main pore diameters to be less than 7 nm, which is in agreement with the shape of the hysteresis loop above (Figure 1(g)). The BET specific surface area and BJH average pore width of the synthesized GO/PVA/Fe_3_O_4_ sample is summarized in Table 1.

### 3.2. Optimization Conditions for Co^2+^ Ion Adsorption onto GO/PVA/Fe_3_O_4_ Nanocomposite

**Figure 2:**
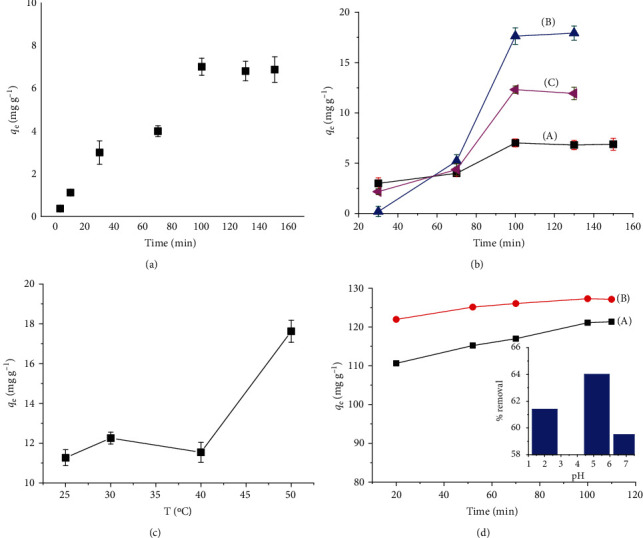
Optimization conditions for adsorption Co^2+^ ion onto GO/PVA/Fe_3_O_4_ nanocomposite: (a) effect of contact time, (b) effect of the adsorbent dosage: (A) 0.03 g; (B) 0.02 g; (C) 0.01 g, respectively, (c) effect of temperature, and (d) effect of pH: (A) pH 2 and (B) pH 5.2 (inserted image: % removal of samples vs. pH).

Figure 2(a) shows that Co^2+^ ion adsorption capacity increased rapidly when contact time was from 3 to 100 minutes, with about 60% of the Co^2+^ removed, and thereafter, the adsorption capacity has a constant trend. The rapid uptake within 100 min was due to the large surface area, the presence of various oxygen functional groups of GO, and PVA that creates an electrostatic interaction with Co^2+^ ion. After that, the adsorption sites of the adsorbent were filled with Co^2+^ ions so the rate of adsorption becomes constant. The contact time here longer than that in other reported materials [36] can be attributed to higher of Co^2+^ initial concentration and lower used adsorbent dose (100 mg·L^−1^, *m* = 0.01 g) as well. The influence of adsorbent dosage was evaluated by changing the mass of GO/PVA/Fe_3_O_4_ adsorbent from 0.0123 g to 0.034 g for treatment of 20 mL of Co^2+^ solution, and the obtained results show that with the increasing mass of the adsorbent, the adsorption capacity decreases and the optimal amount of absorbent is 0.0123 g with an adsorption capacity *q*_e_ is 17.63 mg g^−1^ (Figure 2(b)).  Figure 2(c) reveals that the *q*_e_ increased with the increase in temperature, which suggested that the adsorption of Co^2+^ ion onto the GO/PVA/Fe_3_O_4_ adsorbent may be favored by high temperature and therefore the optimal temperature for this process was selected at 50 C.

The influence of pH on the adsorption process was evaluated with pH change from pH 2 to pH 7 because at higher pH (pH > 7), Co^2+^ ion can be agglomerated as a Co(OH)_2_ precipitate [37, 38]. As shown in Figure 2(d), Co^2+^ ion removal was 61.7% with a *q*_e_ of 121 mg·g^−1^ at pH 2 (curve A) and the removal was about 64.0% with a *q*_e_ of 127.3 mg·g^−1^ at pH 5.2 (curve B), and the UV-Vis spectra at equilibrium time are shown in Figure S2. The obtained results can explain that the high concentration of H^+^ ion (at low pH value) led to the competition between positive charge ions to attach with negative charge oxygen functional groups on GO and PVA. Meanwhile, at higher pH, the concentration of H^+^ ions decreases so there is less competition, and the result is the adsorption increase. Therefore, the optimal pH for adsorption Co^2+^ ion onto GO/PVA/Fe_3_O_4_ was pH 5.2. Effect of K^+^ ion as an interference to the adsorption of Co^2+^ onto GO/PVA/Fe_3_O_4_ was also tested (Table 2). Results show that the presence of K^+^ ion did not interfere with the adsorption efficiency of Co^2+^ ion, even when the concentration of interfering K^+^ ion was 10–80 times higher than the Co^2+^ ion concentration, which is completely consistent with the previous report [39].

### 3.3. The Kinetics of Co^2+^ Ion Adsorption onto GO/PVA/Fe_3_O_4_ Nanoadsorbent

**Figure 3:**
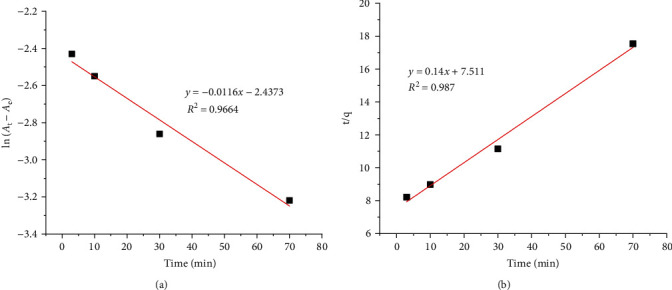
(a) ln(*A*_t_–*A*_e_) vs. *t* plot and (b) *t*/*q* vs. *t* plot to calculate the rate constant. Experimental conditions: *m*_adsorbent_ = 0.034 g, *T* = 30°C, pH = 7, and *C*_o_ = 20 mg·L^−1^.

In this work, two kinetic models including the pseudo-first-order and the pseudo-second-order kinetic models were analysed, which can be represented as follows:

(3) $$\begin{array}{c} \ln\left(q_{e}-q_{t}\right)=\ln\;q_{e}-k_{1}t,\\ \frac{t}{q_{t}}=\frac{1}{k_{2}\cdot q_{e}^{2}}+\;\frac{t}{q_{e}}, \end{array}$$

where *q*_e_ is the adsorption capacity at equilibrium, *q*_t_ is the adsorption capacity at time *t* (min), and *k*_1_ is the pseudo-first-order rate constant of adsorption (min^−1^), and *k*_2_ is the pseudo-second-order rate constant of adsorption (g·mg^−1^·min^−1^). As proved in the previous study [40], equation (2) can be written as follows:

(4) $$\begin{array}{c} \text{ln}\frac{C_{t}}{C_{0}}=\ln\left(\frac{A_{t}-\;A_{e}}{A_{t}-A_{0}}\right)=\;-k_{1}\cdot t, \end{array}$$

where *C*_0_ and *C*_*t*_ are the initial concentration and concentration of Co^2+^ ion in solution, respectively, *A*_o_ and *A*_e_ are the initial and equilibrium absorbance; *k*_1_ was calculated from the slope of the plot of ln (*A*_*t*_ − *A*_*e*_) vs. *t* (Figure 3(a)); and *k*_2_ was calculated from the slope of the plot of *t*/*q*_*t*_ vs. *t*. (Figure 3(b)). Based on comparison of the correlation coefficient (*R*^2^) of two plots, it can be seen that the adsorption of Co^2+^ ion onto the GO/PVA/Fe_3_O_4_ adsorbent was fitted to the pseudo-second-order kinetic model than the pseudo-first-order kinetic model. The rate constant *k*_2_ was fitted at 0.0026 g·mg^−1^·min^−1^.

### 3.4. Investigation of the Thermodynamic Parameters of the Adsorption Process

The thermodynamic parameters of the adsorption process such as enthalpy change (ΔH^0^), entropy change (ΔS^0^), and Gibbs free enthalpy change (ΔG^0^) of reaction are calculated following the second law of thermodynamics, which have been described by the following equations:

(5) $$\begin{array}{c} \Delta G^{0}=\Delta H^{0}\;-\;T\cdot \Delta S^{0}\;=\;-R\cdot T\cdot \;lnK_{c},\\ K_{C}=\;\frac{q_{e}}{C_{e}},\\ \text{ln}K_{c}=\;\frac{-\Delta H^{0}}{R}\cdot \frac{1}{T}+\;\frac{\Delta S^{0}}{R}, \end{array}$$

where *R* is the gas constant (*R* = 8.314 J·mol^−1^·K^−1^), *K*_*c*_ is the equilibrium constant of chemical reaction, and *T* is the absolute temperature (K). ΔH^0^ and ΔS^0^ can be calculated from the slope and the intercept on the plot of ln*K*_*C*_ vs. 1/*T* following equation (4) and the obtained results are given in Table 3. The negative value of ΔG^0^ indicates that the process is spontaneous for all evaluated temperatures. The positive value of ΔH^0^ confirms that the adsorption is endothermic and favors at high temperature. The positive value of ΔS^0^shows the increase in the randomness of the adsorption on GO/PVA/Fe_3_O_4_ for Co^2+^ ion.

### 3.5. Adsorption Isotherm

**Figure 4:**
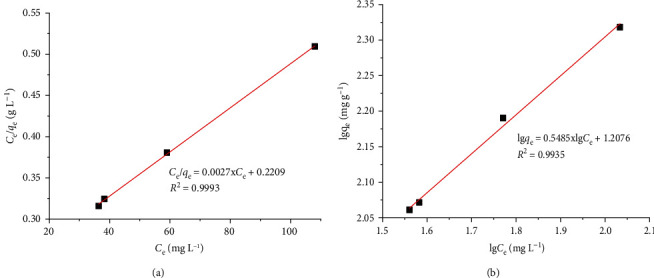
Adsorption isotherm following (a) Langmuir and (b) Freundlich models for Co^2+^ ion adsorption onto the GO/PVA/Fe_3_O_4_, nanocomposite. Experimental conditions: pH = 5.2, *C*_0_ = 100–250 mg·L^−1^, *T* = 303 K, and *m*_adsorbent_ = 0.01 g.

The Langmuir model (equation (5)) assumes a monolayer adsorption onto the homogeneous surface, and there is no transmigration of adsorbate on the surface plane. Meanwhile, the Freundlich model assumes a multilayer adsorption onto the heterogeneous surface (equation (5)).

(6) $$\begin{array}{c} \frac{C_{e}}{q_{e}}=\frac{1}{\left(K_{\text{L}}\cdot q_{\text{max}}\right)}+\;\frac{1}{q_{\text{max}}}\cdot C_{e},\\ \;\mathrm{lg}q_{e}=\mathrm{lg}K_{\text{F}}+\frac{1}{n}\cdot \mathrm{lg}C_{e}, \end{array}$$

where *q*_max_ (mg·g^−1^) is the maximum adsorption capacity of Co^2+^ ion onto the GO/PVA/Fe_3_O_4_ adsorbent; *K*_L,_*K*_F_ are the Langmuir constant and Freundlich constant, respectively; and *n* is a constant. Experimental results following the Langmuir and Freundlich adsorption isotherm are shown in Figure 4, and the fitting of the Langmuir and Freundlich constants is given in Table 4. It can be seen that, with the higher correlation coefficient, the Langmuir model fitted well for the adsorption of Co^2+^ ion onto GO/PVA/Fe_3_O_4_ (Table 4). The maximum monolayer adsorption capacity *q*_max_ is 370.37 mg·g^−1^ and *K*_L_ is 0.0122. Compared to the other adsorbents in the literature for Co^2+^ ion removal (Table 5), the obtained result in our work is so high and impressive, which can be attributed to a very large surface area of GO, facilitating the adsorption process.

### 3.6. Recordation and Regeneration Studies

**Figure 5:**
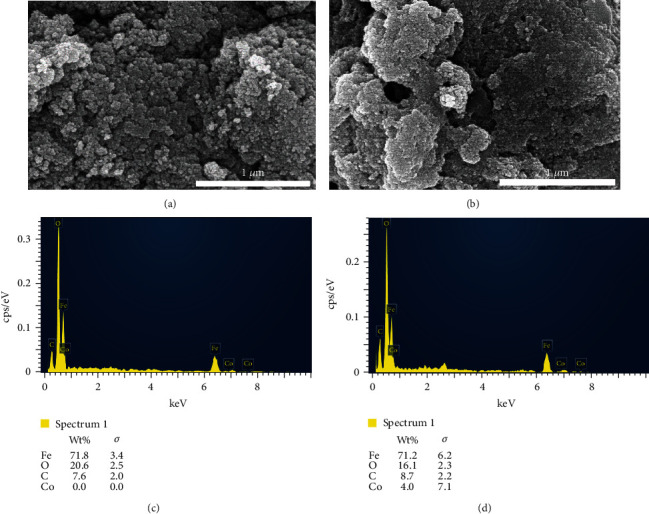
(a, b) SEM and (c, d) EDS spectra of the adsorbent before (a, c) and after (b, d) Co^2+^ ion adsorption process.

SEM images of GO/PVA/Fe_3_O_4_ adsorbent before (Figure 5(a)) and after Co^2+^ adsorption process (Figure 5(b)) are not significantly different excepting several clusters of particles appearing on the surface of adsorbent after the adsorption process, and the surface of the GO/PVA/Fe_3_O_4_ adsorbent after adsorption process is less porous than that before. These observations can be attributed to the presence of adsorbed Co^2+^ ion onto the GO/PVA/Fe_3_O_4_ surface, which was confirmed by the EDS analyses. It can be seen that there was no cobalt element (0 wt.%) on the EDS spectrum of GO/PVA/Fe_3_O_4_ before adsorption (Figure 5(c)); meanwhile, with the sample after Co^2+^ ion adsorption, the cobalt element reached 4 wt.% (Figure 5(d)), in which Co^2+^ ions were adsorbed onto the GO/PVA/Fe_3_O_4_ adsorbent.

As shown in FIgure S3, the regenerated GO/PVA/Fe_3_O_4_ adsorbent can adsorb Co^2+^ ion with only a small decrease in adsorption capacity between the 1st cycle and the 5th cycle. The remaining adsorption efficiency of the 5th cycle was about 86% of the 1st cycle (Figure S3), which implies that the GO/PVA/Fe_3_O_4_ material has a good stability and a high degree of regeneration to use as an excellent adsorbent for removal of Co^2+^ ion in aqueous solution.

## 4. Conclusion

As described in this study, GO/PVA/Fe_3_O_4_ nanocomposite as an effective adsorbent has been simply synthesized via a coprecipitation technique and it was directed to capture Co^2+^ ions from an aqueous solution via an adsorption process. The adsorption process of Co^2+^ ion onto the GO/PVA/Fe_3_O_4_ adsorbent was evaluated by studying the effects of adsorbent dose, the solution pH, and temperature. At optimized adsorption conditions, this process followed the pseudo-second-order kinetic model and the adsorption isotherm was fitted to the Langmuir model with the maximum adsorption capacity to Co^2+^ ion estimated at 370.37 mg·g^−1^. The GO/PVA/Fe_3_O_4_ adsorbent can be regenerated for at least 5 cycles with the remaining adsorption efficiency after the 5th cycle being 86% compared to the first cycle. These findings indicate that the GO/PVA/Fe_3_O_4_ nanocomposite can be considered as a good candidate for the removal of Co^2+^ ion from aqueous solutions.

## Figures and Tables

**Table 1:** BET surface area analysis of as-synthesized GO/PVA/Fe_3_O_4_ sample.

Surface area (m^2^·g^−1^)			BJH pore volume (cm³·g^−1^)	BJH average pore width (nm)
Langmuir surface area	BET surface area	BJH pore area		
122.328 ± 3.367	82.071 ± 0.163	97.218	0.137 ÷ 0.144	5.931 ÷ 6.725

**Table 2:** The effect of K^+^ as an interfering ion to the adsorption of Co^2+^ ion onto GO/PVA/Fe_3_O_4_ nanocomposite^(^^*∗*^^)^.

Interfering ion	Fold ratio	Adsorption efficiency (%)
K^+^	10	63.7
K^+^	20	61.9
K^+^	50	62.3
K^+^	80	63.0

^(^^*∗*^^)^Experimental conditions: *C*_o_ = 100 mg·g^−1^, pH = 5.2, *T* = 25 C, and *m*_adsorbent_ = 0.01 g.

**Table 3:** Thermodynamic parameters of the adsorption Co^2+^ ion on GO/PVA/Fe_3_O_4_.

ΔG^0^ (kJ·mol^−1^)				ΔH^0^ (kJ·mol^−1^)	ΔS^0^ (J·mol^−1^·K^−1^)
298 K	303 K	313 K	323 K	22.04	106.47
−9.8	−10.07	−10.06	−12.38	—	—

**Table 4:** The Langmuir and Freundlich isotherm parameters.

Langmuir isotherm			Freundlich isotherm		
*q*_max_ (mg·g^−1^)	*K*_L_	*R* ^2^	*K*_F_	*N*	*R* ^2^
370.37	0.0122	0.9993	15.8767	0.5485	0.9935

**Table 5:** Comparison of various adsorbents for Co^2+^ ion removal.

Adsorbent materials	*q*_max_ (mg·g^−1^)	Ref.
Palygorskite	8.88	[41]
Lignocellulose/montmorillonite	93.43	[42]
Fe_3_O_4_/bentonite	18.76	[43]
Hydroxyapatite	22.50	[44]
GO/PVA/Fe_3_O_4_	370.37	This work

## Data Availability

The data used to support the findings of this study are included within the article.

## Supplementary Materials

Supplementary Materials Figure S1(a) UV-Vis spectra of various concentrations of Co^2+^ solution and (b) the calibration curve for Co^2+^ concentration measurement. Figure S2The UV-Vis spectra of samples at different pH values vs. equilibrium time: (i) pH 2 and (ii) pH 5.2. Experimental conditions: adsorbent dose 0.01 g, initial concentration of Co^2+^ ion 100 mg·L^−1^, and temperature 25°C. Figure S3GO/PVA/Fe_3_O_4_ regeneration results. Conditions: adsorbent dose 0.01 g, initial concentration of Co^2+^ ions 100 mg·L^−1^, temperature 25°C, and pH = 5.2.
