# Supplementary material for: Graphene Oxide/Polyvinyl Alcohol/Fe3O4 Nanocomposite: An Efficient Adsorbent for Co(II) Ion Removal
Source: J Anal Methods Chem. 2021 Mar 9;2021:6670913. doi: 10.1155/2021/6670913 (PMC7964109; doi:10.1155/2021/6670913)
Supplement: Supplementary Materials — Figure S1(a) UV-Vis spectra of various concentrations of Co2+ solution and (b) the calibration curve for Co2+ concentration measurement. Figure S2The UV-Vis spectra of samples at different pH values vs. equilibrium time: (i) pH 2 and (ii) pH 5.2. Experimental conditions: adsorbent dose 0.01 g, initial concentration of Co2+ ion 100 mg·L−1, and temperature 25°C. Figure S3GO/PVA/Fe3O4 regeneration results. Conditions: adsorbent dose 0.01 g, initial concentration of Co2+ ions 100 mg·L−1, temperature 25°C, and pH = 5.2. [file 6670913.f1.docx]

*Supplementary Information*

**Graphene Oxide/Polyvinyl alcohol/Fe_3_O_4_ nanocomposite- An Efficiency Adsorbent for Co(II) Ion Removal**

Thu D. Le, Luyen T. Tran, Hue T. M. Dang, Huyen T. T. Tran, Hoang V. Tran^♣^

*School of Chemical Engineering, Hanoi University of Science and Technology, 1 Dai Co Viet Road, Hanoi, Vietnam*


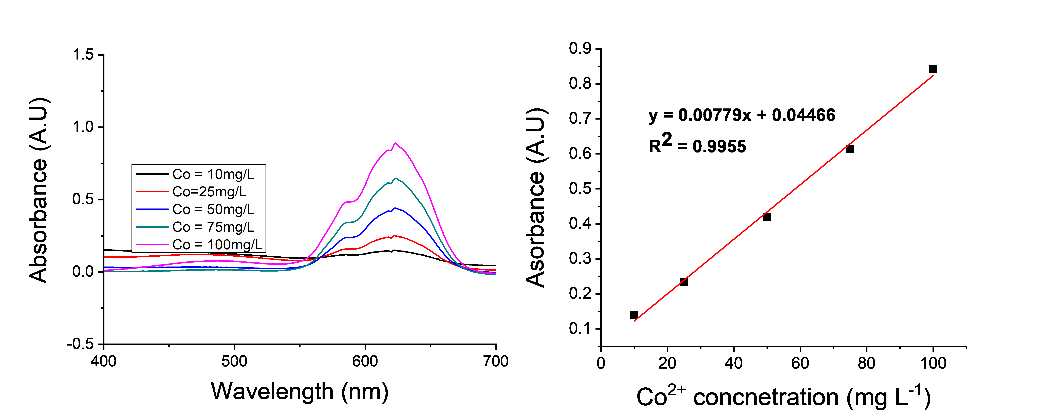

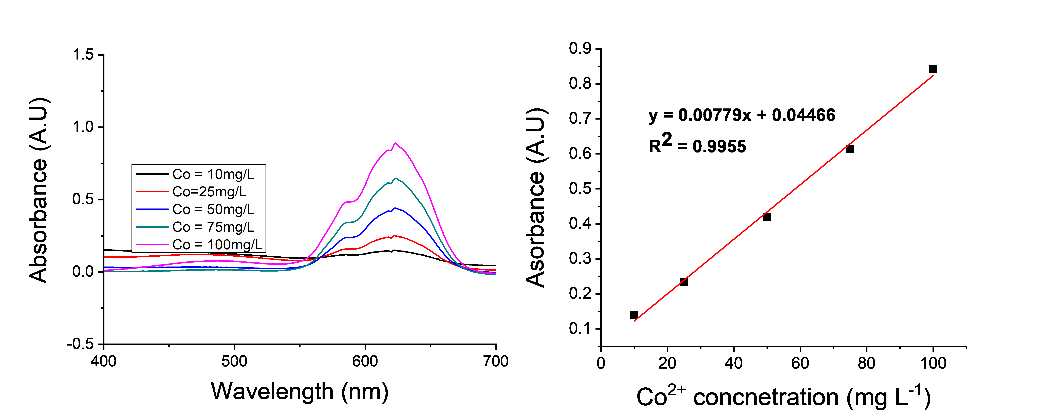


(b)

(a)

***Figure SI.1.*** (a) UV-Vis spectra of various concentrations of Co^2+^ solution and (b) the calibration curve for Co^2+^ concentration measurement.


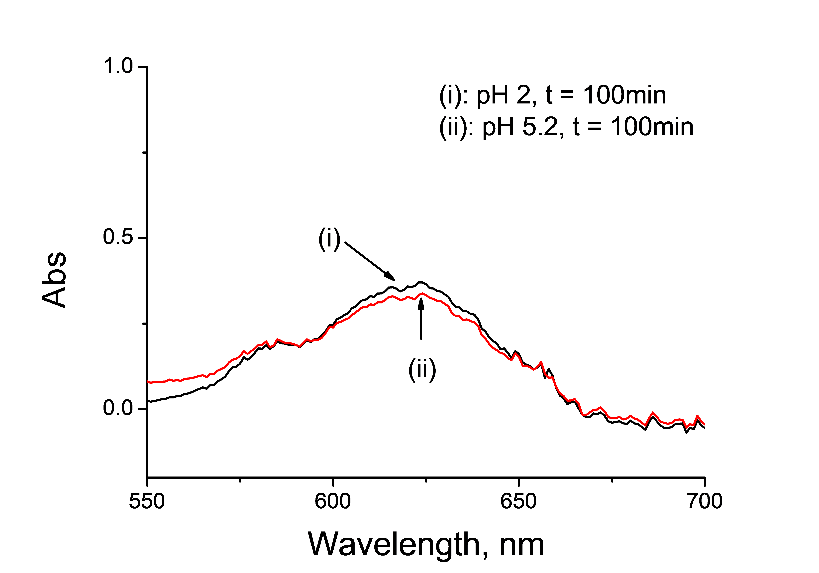


***Figure SI.2.*** The UV – Vis spectra of samples at different pH vs. equilibrium time: (i) pH 2 and (ii) pH 5.2. Experimental conditions: adsorbent dose 0.01 g, initial concentration Co^2+^ ion 100 mg L^-1^, temperature 25 ^0^C

***
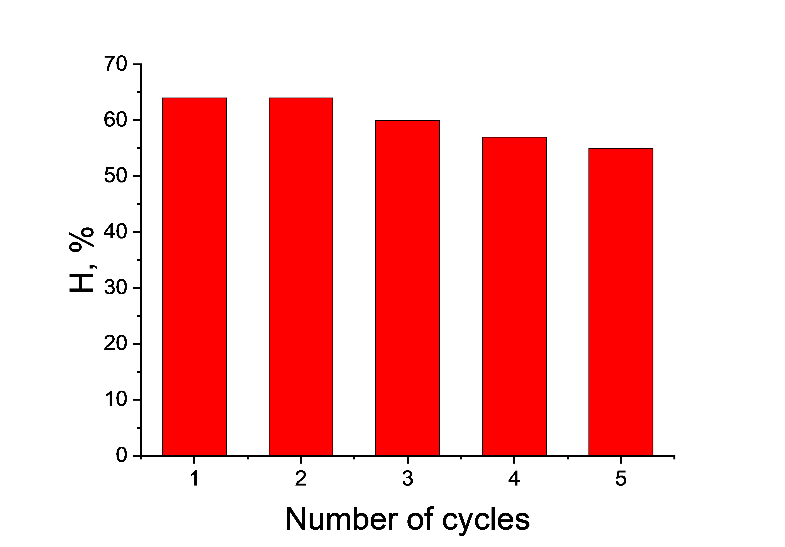
***

***Figure SI.3.*** GO/PVA/Fe_3_O_4_ regeneration results. Conditions: adsorbent dose 0.01 g, initial concentration Co^2+^ ions 100 mg L^-1^, temperature 25 ^o^C, pH = 5.2.
